# Exploiting passive behaviours for diverse musical playing using the parametric hand

**DOI:** 10.3389/frobt.2024.1463744

**Published:** 2024-12-13

**Authors:** Kieran Gilday, Dohyeon Pyeon, S. Dhanush, Kyu-Jin Cho, Josie Hughes

**Affiliations:** ^1^ CREATE-Lab, Department of Mechanical Engineering, Swiss Federal Technology Institute of Lausanne (EPFL), Lausanne, Switzerland; ^2^ Department of Mechanical Engineering, Soft Robotics Research Center, Institute of Advanced Machines and Design (IAMD), The Institute of Engineering Research at Seoul National University, Seoul, Republic of Korea

**Keywords:** musical robots, embodied intelligence, robotic manipulation, soft manipulation, anthropomorphic hands, expressive playing

## Abstract

Creativity and style in music playing originates from constraints and imperfect interactions between instruments and players. Digital and robotic systems have so far been unable to capture this naturalistic playing. Whether as an additional tool for musicians, function restoration with prosthetics, or artificial intelligence-powered systems, the physical embodiment and interactions generated are critical for expression and connection with an audience. We introduce the parametric hand, which serves as a platform to explore the generation of diverse interactions for the stylistic playing of both pianos and guitars. The hand’s anatomical design and non-linear actuation are exploitable with simple kinematic modeling and synergistic actuation. This enables the modulation of two degrees of freedom for piano chord playing and guitar strumming with up to 6.6 times the variation in the signal amplitude. When only varying hand stiffness properties, we achieve capabilities similar to the variation exhibited in human strumming. Finally, we demonstrate the exploitability of behaviours with the rapid programming of posture and stiffness for sequential instrument playing, including guitar pick grasping. In summary, we highlight the utility of embodied intelligence in musical instrument playing through interactive behavioural diversity, as well as the ability to exploit behaviours over this diversity through designed behavioural robustness and synergistic actuation.

## 1 Introduction

Musical instruments serve as tools and assist for musical thinking within the cognitive loops of musicians’ brains (Aho, 2016). The exploitation of such a tool through physical interactions is a critical part of this process, and it must be the guiding principle behind robotic musicianship (Weinberg et al., 2020). Music-playing robots are sought after for a variety of reasons, including pure entertainment (Jeon, 2017; Li and Lai, 2014; Yang et al., 2023), restoration or extension of human functionality (Larsen et al., 2013; Leigh and Maes, 2018), and as a medium for exploring robot–environment interactions (Hughes et al., 2018; Wang et al., 2022; Zhang et al., 2011). However, the majority of these systems are bespoke designs optimised for a particular instrument and, often, a particular “robotic” playing style (Weinberg et al., 2020). A more general-purpose system based on human-like interactions (Gilday et al., 2023a) may be able to form more emotional connections during entertainment, restore more expressive playing to prosthesis users, and deepen our understanding of interactions.

Developing manipulators with dexterity and variable stiffness is an enabling technology for robotic music playing. Due to the complexity of both hardware and control systems, these types of dexterous robotic hands have seen limited practical use (Gilday et al., 2023b). Soft manipulation is an emerging field attempting to exploit embodied intelligence in order to develop more capable and adaptive hardware without creating additional bottlenecks in control systems (Hughes et al., 2016; Puhlmann et al., 2022). This approach has been applied to piano playing with a passive hand able to generate diverse progressions with sequential soft interactions (Hughes et al., 2018). However, design and the corresponding exploitation of complex soft-body interactions is an unsolved problem (Mengaldo et al., 2022), especially when designing for more than one interaction mode (Wang and Iida, 2023; Catalano et al., 2014).

**FIGURE 1:**
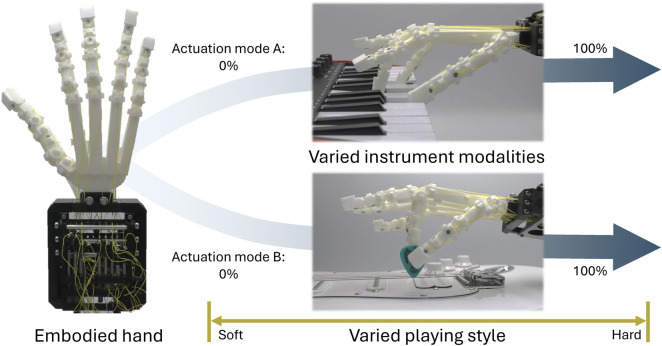
System overview. The parametric hand utilises carefully designed interaction behaviours and low degree-of-freedom control for the online control of the musical instrument and playing style modalities.

Using the parametric hand—a hand developed for customisation of up to 20 degree-of-freedom (DoF) interactions through parametric design, rapid prototyping, and synergistic actuation—we hypothesise that embodied intelligence within manipulators allows for diverse, multi-instrument modalities and variable playing styles with simple control. Figure 1 outlines this concept, where two actuators control the parametric hand posture in two synergies, allowing keyboard chord playing and guitar strumming while holding a pick. Additionally, the degree of actuation modulates the open-loop interaction forces with non-linear series elasticity for variations in stylistic playing.

To highlight the controllable behaviours of the hand, we use a simple kinematic model and configure the tendons’ series elasticity to generate finger key press stiffness of 0.03–0.1 N/mm and guitar pick plucking stiffness of 0.06–0.2 N/mm. We then observe the change in playing style on both piano chords and guitar strumming via this modulation. We detect 87% variation in piano chord signal amplitude, limited when compared to human playing, but up to 560% variation in strumming amplitude, which covers 65% of the dynamic range compared with human strumming.

Overall, the behaviour programmability and exploitability are demonstrated using simple kinaesthetic teaching procedures and online control of instrument and playing styles, which are enabled by the robustness of hand behaviours and low DoF control. Therefore, we conclude that embodied intelligence is critical for more creative and less “robotic” musical instrument playing. Additionally, a hand designed using this approach is more readily exploitable by existing AI techniques and generative models for manipulation and composition.

The remainder of this work is structured first into methods, where the hand design and kinematic model are explained. The experimental setup then details the procedure for stiffness and playing style testing, whose results are presented in the following section. The paper concludes with a demonstration of both instruments being played by the same hand, and finally, a discussion including the limitations and future directions.

## 2 Methods and materials

In this section, we introduce the parametric hand and the process for designing and tuning active and passive behaviours.

### 2.1 Parametric hand

The parametric hand is a single-piece 3D printable hand with anthropomorphic rolling contact joints and tendon layout. The hand in this work is fabricated through SLS printing using polyamide (PA2200). However, it is also suited for FDM printing with nylon- or polypropylene-like materials to create high stiffness bones and thin, tough, living hinge ligaments that constrain joint motions with minimal resistance. The hand exhibits complex behaviours due to non-linear joint moment arms (Gilday et al., 2023a), five tendons per four DoF finger (Pollard and Gilbert, 2002) (plus sixth thumb tendon for increased opposition force), and frictional/sequential interaction effects (Hughes et al., 2018). Additionally, the dislocatable joints and series elasticity in the tendon routing improve manipulation robustness and behavioural stability when actuated with synergies (Catalano et al., 2014).

**FIGURE 2:**
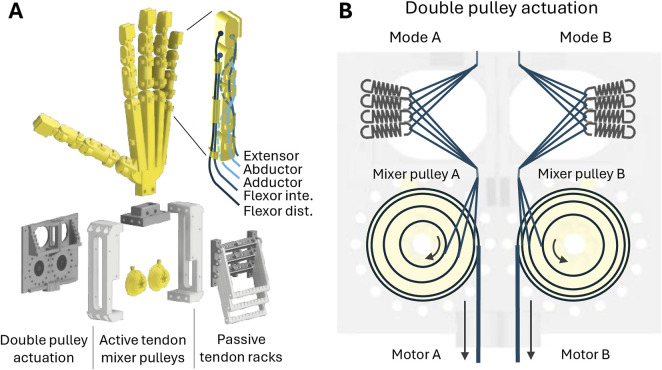
Parametric hand design. **(A)** Exploded view of the hand and modular actuation system. Two independent actuation modes can be configured with up to 5 independent active tendons (or 10+ non-independent tendons) and 30 passive tendons. Each finger has five tendons for four DoF controls. **(B)** Active tendon routing through series, non-linear elasticity and up to five-to-one mixer pulley.

Figure 2 shows the design and actuator configuration of the hand. The parametric hand is based on human skeleton proportions, which were determined through a direct measurement of bone lengths and joint diameters in a real hand, and the double pulley actuation system is designed to drive two motor synergies independently. Figure 2A shows the exploded design of the hand and actuation system. The hand is generated through parametric design in OpenSCAD^1^ and has the potential for full control of all four DoFs per finger. The actuation system consists of two parts: a passive tendon unit, where non-active tendons are anchored to a series of racks via springs, with adjustable stiffness, and an active tendon unit, where tendons are actuated in synergies by mixer pulleys with multiple radii. Figure 2B shows the routing of active tendons: first, through a non-linear series elastic stage and then to mixer pulleys, where synergies are defined by the type of tendon and relative pulley size. The mixer pulleys are actuated by a single tendon, which can be driven through Bowden tube actuation or directly by motor connection.

For the application of music playing, we selected two desirable postures to base the synergistic actuation on. For piano playing, we selected a common A minor chord. This requires some dexterity with the use of three fingers. For guitar playing, we choose a posture for grasping a guitar pick, which can be used for strumming, requiring the precision and application of force at the fingertips.

### 2.2 Kinematic model

**FIGURE 3:**
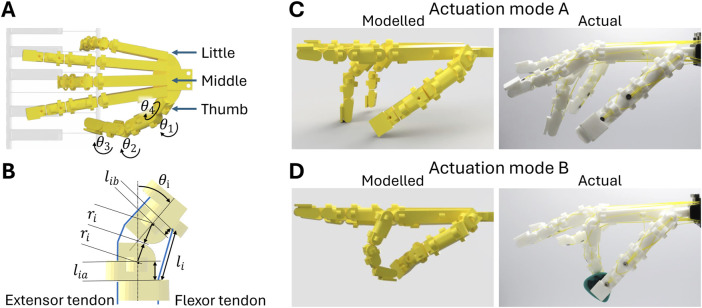
Actuation modes and finger kinematics. **(A)** Manual positioning to calculate mixer pulley radii. **(B)** Relevant joint geometries for computing tendon–joint angle relationships. **(C)** Actuation mode A (piano chord) modelled and actual posture. **(D)** Actuation mode B (guitar pick grasping) modelled and actual posture.

Figure 3 shows the process for determining actuation modes for the hand. Figure 3A shows an example posture manually modelled in Autodesk Fusion 360. For piano chord playing, the thumb, middle, and little fingers are positioned similar to a human’s posture, and a keyboard overlay ensures correct finger spacing. Figure 3D depicts a guitar strumming posture, in which the thumb and index finger are positioned to pinch a pick modelled from human strumming examples.

With the posture known, the required actuation can be calculated from the joint angles. A combination of five tendons per finger with no slack maps to a unique set of joint angles, $\theta_{1}$ – $\theta_{4}$ , via the finger’s joint geometries. Figure 3B shows the relevant geometries used to calculate the change in the length of the extensor and flexor tendons to the change in the joint angle $\theta_{i}$ relative to a fully extended starting posture, as mentioned in Gilday et al. (2020). The calculated change in the length of the tendons was used to distinguish between passive and active tendons; extending tendons can be connected passively, where retracting tendons must frequently be actuated. The passive tendons are connected to linear springs with spring constant tuned for desired behaviours. The active tendons are routed to a mixer pulley whose relative diameters are based on the change in the length of the active tendons.

### 2.3 Tendon configuration

For both actuation modes shown in Figures 3C, D, the kinematic model acts as a starting point to achieve the desired postures. The tendon configuration requires tuning in order to ensure desired interaction behaviours. This is currently achieved manually by varying the pre-tension and stiffness of each tendon.

Table 1 shows the final tendon configuration used. Pulley A contracts the thumb, middle, and little finger flexors to form a chord-playing posture (Figure 3C). Additionally, the thumb abductor is routed through a low stiffness, series elastic spring to the smallest radius on pulley A. This serves to increase that tendon stiffness with the actuation of pulley A, increasing resistance to adduction force when pressing a key with the side of the thumb and not interfering with the second designed behaviour mode. The actual posture shown in Figure 3C differs from the modelled posture; the PIP joints rotate less than the modelled posture due to high friction and compliance in the tendon paths.

**TABLE 1:** Tendon configuration. Actuation mode A/B (and pulley diameter (mm)) or passive spring stiffness (N/mm).

	Finger				
Tendon	Thumb	Index	Middle	Ring	Little
Flx. Dist	A (ϕ11.6) , B (ϕ23.8)	B (ϕ31.2)	A (ϕ18.0)	0.32	A (ϕ14.8)
Flx. Inte	A (ϕ11.6) , B (ϕ23.8)	B (ϕ21.7)	A (ϕ18.0)	0.32	A (ϕ14.8)
Extensor	0.32	0.87	0.32	0.32	0.32
Abductor	0.11, A (ϕ11.6)	0.32	0.11	0.11	0.11
Adductor	0.32	0.32	0.11	0.11	0.11
Opposition	0.32	—	—	—	—

The modelled posture for mode B intersects the thumb and index finger to ensure a net pinching force, as shown in Figure 3D. Pulley B contracts the thumb and index finger flexors; the asymmetric stiffness of the abductor and adductor allows some abduction as the PIP joint is flexed and increases tension in both tendons. Spring stiffness for passive tendons is chosen from trial and error. There is room for customisation as higher stiffness increases joint stability, allowing for higher interaction forces while also simultaneously requiring stronger actuation to overcome antagonism. The index finger passive tendons have higher stiffness to increase resistance to adduction forces from the thumb when pinching a guitar pick. Similar to mode A, the actual posture (Figure 3D) shows less rotation in joints towards the fingertip. However, successful guitar pick grasping is observed.

### 2.4 Experimental setup

**FIGURE 4:**
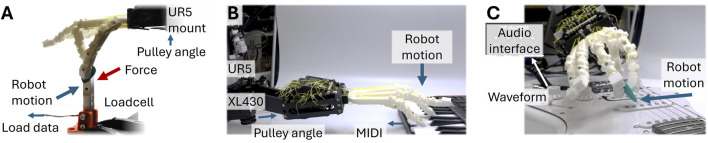
Experimental setup. **(A)** Stiffness variation experiment measuring load cell force with robot displacement. **(B)** Piano playing style experiment with MIDI signals recorded and pulley A angle varying. **(C)** Guitar playing style experiment with waveforms recorded via an audio interface and pulley B varying pick grasping force.

Figure 4 shows the experimental setup for measuring stiffness change and recording instrument interactions. As shown in Figure 4A, the hand is mounted on a UR5 arm to control and track position, and a load cell is fixed to the table. Load and displacement are measured when pushing the load cell with varying actuation degrees. Hand actuation is achieved using two XL430-W250-T Dynamixel servos for precise mixer pulley angle control. Each test, varying the two actuation mode angles, is repeated three times.

Figures 4B, C show the setup with the hand mounted on the UR5 arm with either the piano or guitar placed on the table. For the piano (Akai Professional MPK Mini MK3 with velocity-sensitive keys), MIDI signals are read and converted to waveforms via the direct digital interface. Robot trajectories for key pressing are recorded with kinaesthetic teaching (physically guiding the robot). For the electric guitar (Dimavery ST-312), signals are read via an audio interface (M-Audio M-Track Duo), and the robot strumming motion is a single linear movement. Three repetitions are performed with each instrument and actuation angle, while the robot’s playing motion is fixed.

## 3 Results

### 3.1 Stiffness range

In the first set of results, we characterise the stiffness change in the fingers when pressing in the same axis as a piano key and the stiffness of the combined guitar pick and pinching fingers.

**FIGURE 5:**
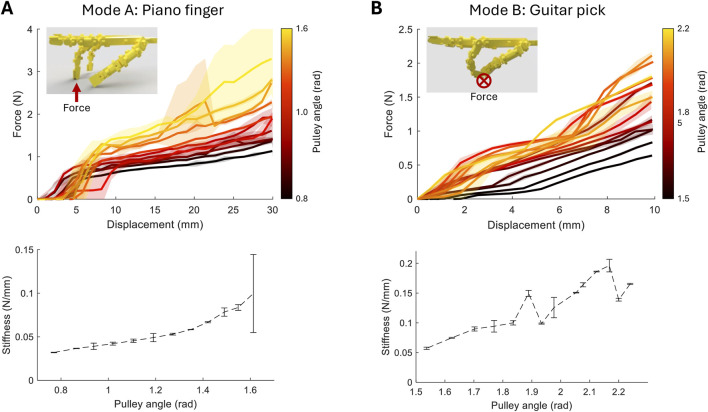
Stiffness experiment results in mean and standard deviation. **(A)** Middle finger force/displacement graph with the varying pulley A angle (upper) and computed average stiffness (lower). **(B)** Grasped pick force/displacement graph with the varying pulley B angle (upper) and computed average stiffness (lower).

Figure 5A shows the force displacement graphs of the middle finger when varying the actuation of pulley A from the initial formation of the posture to the maximum angle found for successful key pressing. The force increases gradually with the increased actuation. Figure 5A (lower) shows that extracting the mean stiffness results in a total stiffness increase from 0.032 to 0.100 N/mm. At the highest actuation angles, the key pressing begins to break down. Small variations in the starting posture can cause the fingers to slip and lose contact with the load cell, resulting in a significantly higher standard deviation. Previous studies suggest that a real human finger has a stiffness range of 0.3–3 N/mm (Hajian and Howe, 1997). The stiffness range can be shifted through parametric design by increasing joint diameters or tendon spring constants. However, increasing the dynamic range requires the exploitation of more non-linear effects or the actuation of more than one tendon, including the extensor tendons, which provide a stabilising effect on the finger.

Figure 5B shows the guitar pick stiffness results. The force–displacement profile is more linear. Pick deflection causes displacement more than finger motion; therefore, static friction in the tendon routing has a lesser effect. Applying a force to the pick levers the fingers apart. Therefore, the higher the stiffness of the fingers the greater the pinching force and the more force exerted on the load cell. This is observed in the results with an overall trend of stiffness increasing from 0.057 to 0.166 N/mm. Both actuation modes exhibit open-loop stiffness control with dynamic range ≈ 3. During testing, the pick is realigned with the load cell for each test. This introduces the variance shown in Figure 5B, especially if relative orientation changes. However, the overall correlation remains positive.

It is not clear how critical the accuracy of stiffness control is in expressive music playing. Indeed, focus on accuracy may lead to the maligned “robotic” playing. Inaccuracy shows potential to be compensated through the selection of motion, interactions, feedback, and learning. This is demonstrated by the choice of test axes and procedures mentioned above, which result in greater stiffness control accuracy with the piano finger than that with the guitar pick. However, observing the stiffness characteristics can aid us in understanding the dynamic range of playing styles and influence the next generation of hand design and actuation.

### 3.2 Piano playing

**FIGURE 6:**
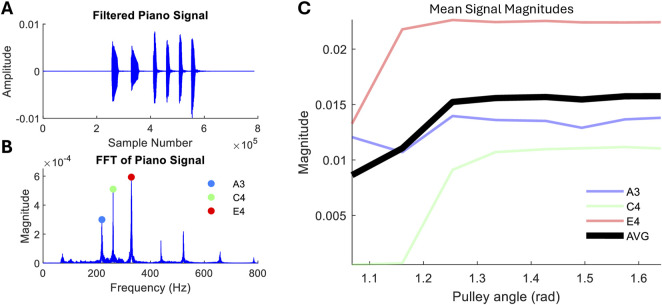
Piano playing experiment. **(A)** Example of the recorded signal playing a chord six times. **(B)** Frequency analysis, with first harmonics labelled. **(C)** Individual key mean first harmonic amplitude and average of all keys against the pulley A angle.

Figure 6 shows the results of the piano key-playing experiment. In each test, the A minor chord that consists of A3, C4, and E4 is played six times. An example signal is shown in Figure 6A. A fast Fourier transform (FFT) is performed on this (Figure 6B), allowing the isolation of the first harmonics of each key press.

Three repeats are performed for each pulley A angle, and the mean first harmonic magnitude is plotted in Figure 6C. In this study, we observe a significant increase in amplitude for the first 0.2 radians before magnitude plateaus. This is likely due to the mismatch in key and finger stiffnesses and velocity, which becomes the limiting factor. With high relative finger stiffness, increasing stiffness will not result in faster key pressing, but only increasing robot speed will. Despite this saturation, we see an overall increase in the average magnitude of 87%.

The increase in note amplitudes is most significant in the middle and little fingers, C4 and E4, respectively (Figure 6C). The amplitude of the note played by the thumb shows much lower variation. This is due to the thumb forces being directed to the abductor tendon rather than the flexors, which has a less straightforward influence on thumb forces due to the coupling with the PIP joint and smaller MCP moment arm.

**FIGURE 7:**
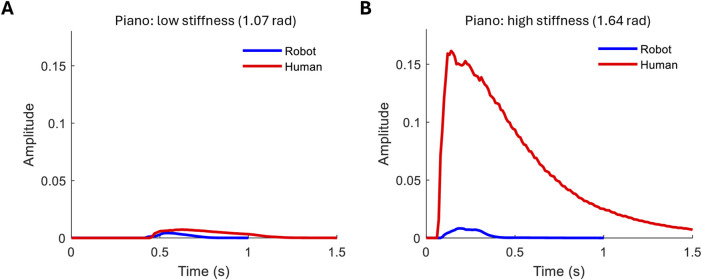
Comparison between robot and human piano playing. **(A)** Low-stiffness playing style. **(B)** High-stiffness playing style.

Figure 7 shows the comparison between the robot hand chord playing and a human instructed to play lightly (Figure 7A) and strongly (Figure 7B). The hand is able to match the light playing, although the strong human playing reaches an order of magnitude above the robot playing. Although the individual finger stiffness has a lower dynamic range than a human finger (Figure 5A), the reduced range here is also due to extraneous factors such as the limited velocity of the robot and additional factors of human playing such as wrist compliance.

### 3.3 Guitar playing

**FIGURE 8:**
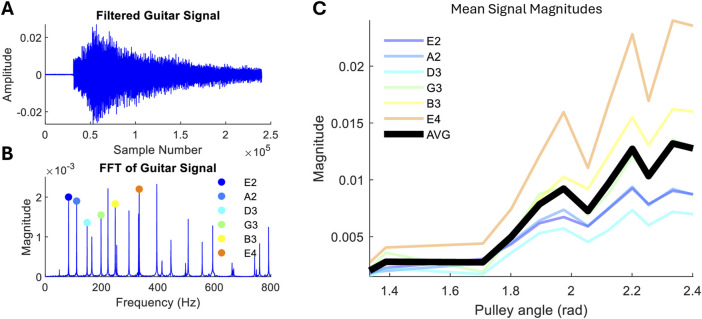
Guitar playing experiment. **(A)** Example of the recorded signal strumming six strings once. **(B)** Frequency analysis, with first harmonics labelled. **(C)** Individual string mean first harmonic amplitude and average of all strings against the pulley B angle.

The results of performing the same frequency analysis as the piano playing but for guitar strumming are shown in Figure 8. In each test, the guitar was played in open tuning with all six strings unfretted. We observe that increasing the pulley B angle leads to a significant increase in the signal amplitude. Across all strings, amplitudes are correlated with high variance over the actuation degree (Figure 8C). The variance is likely due to inaccuracies in open-loop stiffness control shown in Figure 5B, which originates from uncertainties in hand posture and relative pick position. As with the stiffness results, the trend remains positive, and the overall change in the average magnitude is 560%.

**FIGURE 9:**
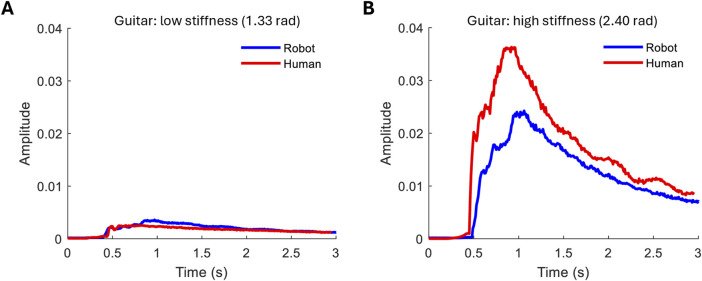
Comparison between robot and human guitar playing. **(A)** Low-stiffness playing style. **(B)** High-stiffness playing style.

The high magnitude variation detected in signals from strumming with different hand stiffness corresponds more closely to the variation seen in human playing than to piano playing. Figure 9 compares the robot to a human playing when instructed to play as lightly and strongly as possible while maintaining a similar strumming speed. At the tested speed, the hand is able to cover 65% of the dynamic range in human-playing amplitude.

### 3.4 Behavioural robustness

One criterion for embodied intelligence in hands is the robust and exploitable behaviours with minimal control inputs. For chord playing and guitar strumming with repetitive playing motions, successful playing is observed for a large range of actuation inputs. As shown in Figure 6C, playing of each key is successful over the pulley range of 1.07–1.64 radians, i.e., input can vary up to 50% before keys are not pressed with enough force ( <1.07 rad) or the fingers miss or fail to release keys ( >1.64 rad). Similarly with strumming (Figure 8C), each string can be played successfully when varying the actuation from 1.33 to 2.40 rad (input range 80%). Beyond this range, playing breaks down, and either the guitar pick slips from the grasp ( <1.33 rad) or the fingers collide with strings ( >2.40 rad), requiring revised robot trajectories.

### 3.5 Combined playing

**FIGURE 10:**
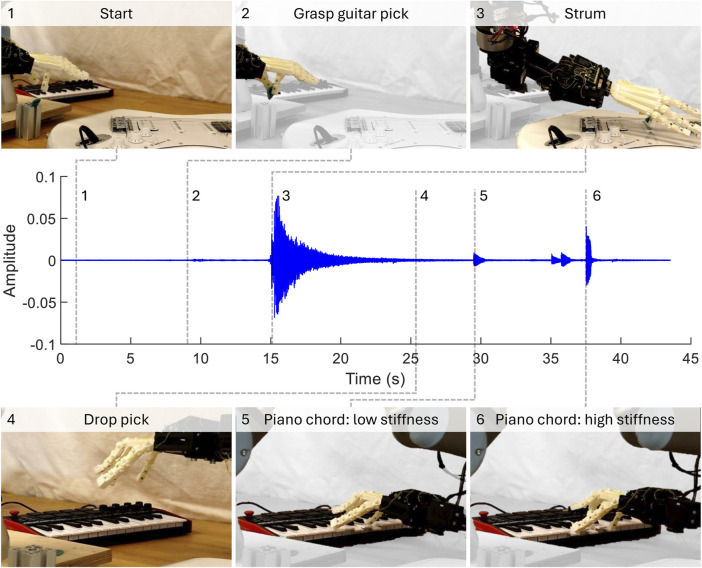
Multi-instrument, multi-style demonstration. With kinaesthetic teaching and online control of two actuators, the robot can grasp a pick, strum the guitar, and then play piano chords with varying styles.

The independent actuation modes allow for real-time variation in both the playing style and instrument. Figure 10 shows a sequence of the hand grasping a pick, strumming the guitar, releasing the pick, and finally playing a sequence of piano chords. With simple kinaesthetic teaching and online playing variation with only two actuation inputs, diverse sequences can be programmed rapidly.

During sequence programming and replaying, some weaknesses of this system were observed. Although robustness in strumming and chord playing are demonstrated relative to the actuation and minor variations in starting posture, less robustness is observed in other actions or some external inputs. For example, pick grasping is highly sensitive to starting hand posture and relative placement of the pick on the table (Figure 10 start). Additionally, small variations in the piano chord playing trajectory or relative keyboard position might result in the wrong key being pressed, especially if the fingers are deflected during playing. There is potential to increase robustness through design, for example, by increasing chord playing middle and little finger abduction/adduction stiffness. However, the stricter the set of requirements, the more complex the design becomes and more work is required to understand the behaviour space of this hand and general-purpose hands overall (Gilday and Iida, 2022).

## 4 Discussion and conclusion

By tuning the actuation modes of the parametric hand for multi-instrument and multi-playing styles, which are robust to significant changes in actuation inputs, we demonstrate the capabilities of embodied intelligence for music playing. There are limitations to this preliminary study, especially when comparing playing directly to human examples without a method for isolating passive finger stiffness. However, the results remain significant from the perspective of developing exploitable, emergent behaviours with a customisable hand design. In particular, the ability to exploit varying playing styles with one degree of freedom greatly simplifies control for rapid programming. Additionally, with focus on intelligent behaviours comes increased ability to explore interesting interactions, varying playing styles with different speeds (Figure 10) or sequential interactions (Hughes et al., 2018).

Although signal amplitude simplifies playing style, the variation exhibited in guitar strumming and piano playing when only varying a single input shows a significant portion of the variation seen in human playing (65% for guitar). Additional work can be done to maximise stiffness variation through parametric design. However, gradually integrating the hand with increasingly advanced features will improve performance to a greater degree at the risk of creating additional bottlenecks in control and learning. The parametric hand aims in improving baseline performance, allowing for more diverse instrument playing through advanced actuation (additional active tendons to increase stiffness range and additional actuated synergies for the modulation of new behaviours, variable arm motions, and wrist compliance). This leads to more naturalistic and expressive instrument playing, rather than “robotic” instrument playing.

The multi-functionality and versatility of the parametric hand can be transferred to other behaviours, such as one actuation mode as a precision pinch grasp and a second mode as a power enveloping grasp (Feix et al., 2015). There is also potential for the extension of behaviours, with additional actuation modes added in parallel or series. Flexor tendons, in particular, can be parallelised since the active actuation mode is dominant, as demonstrated in the thumb flexors (Table 1). Therefore, only modes that involve pulling tendons can scale. However, any mode which controls the release of tendons (needed for more complex or precise postures) will interfere with other actuation modes and needs careful design or an alternative mechanism. Additionally, there are limitations to the hand design and control of behaviours, which need to be overcome. Compared to a human finger, the stiffness magnitude and range is low, and to what extent this can be solved through design needs further study. The addition of a skin is essential for interactions more dependent on contact friction. Finally, we foresee challenges in defining and tuning hand behaviours for more complex interactions as the current approach of inverse kinematics and stiffness tuning is unlikely to be sufficient.

The parametric hand has a significant future potential for integration with artificial intelligence to control robot motions and musical composition. With reduced control dimensionality, reinforcement learning and other approaches are readily applicable. In particular, approaches allowing for emergent interactions are exciting for exploiting the hand’s capabilities and investigating compositions with varying designs beyond purely anthropomorphic hands.

## Data Availability

The datasets presented in this study can be found in online repositories. The names of the repository/repositories and accession number(s) can be found at: https://zenodo.org/uploads/12698245.

## Footnotes

1
https://github.com/kg398/100_fingers/tree/release_v1.0

## Supplementary material

SUPPLEMENTARY VIDEO S1 Video overview showing playing style experiments and combined playing example.
